# Diabetes and Cancer-Pathophysiology and Clinical Implications: A Systematic Review

**DOI:** 10.3390/cimb48101086

**Published:** 2026-10-20

**Authors:** Zahid Hasan, Zinnat Ara Moni, Tasnima Kamal, Md Matakabbir Hossain, Yearul Kabir, Farhadul Islam

**Affiliations:** 1 Department of Biochemistry and Molecular Biology, University of Rajshahi, Rajshahi 6205, Bangladesh; zahidhasan.bmb.ru@gmail.com (Z.H.); zinnatara.edu@gmail.com (Z.A.M.); rakatasnima2015@gmail.com (T.K.); matakabbirhossain@gmail.com (M.M.H.); 2 Department of Biochemistry and Molecular Biology, University of Dhaka, Dhaka 1000, Bangladesh; ykabir@yahoo.com; 3 School of Medicine and Dentistry, Griffith University, Gold Coast Campus, Southport, QLD 4222, Australia

**Keywords:** cancer pathogenesis, hyperglycemia, chronic inflammation, metabolic disorder, insulin resistance, metformin, unfolded protein response (UPR)

## Abstract

Diabetes Mellitus and cancer are major global health problems with rising incidence worldwide. This systematic review was conducted following PRISMA 2020 guidelines by searching PubMed, Scopus, ScienceDirect, SpringerLink, and Google Scholar for studies published between 2015 and 2026. Studies investigating the association between diabetes mellitus and cancer were selected based on predefined criteria, and data were synthesized using a narrative approach. The findings indicate a strong and mechanistically complex association between diabetes mellitus and multiple cancers, particularly pancreatic, breast, colorectal, and prostate cancers. Hyperglycemia, insulin resistance, and chronic inflammation are consistently implicated in tumor initiation and progression through key signaling pathways, including Wnt/β-catenin, PI3K/AKT/mTOR, and AMPK. A bidirectional relationship is most evident in pancreatic cancer, highlighting its unique metabolic and oncogenic interplay. Shared molecular mediators such as cytokines, microRNAs, and metabolic regulators further support a common pathogenic link. Metformin, an antidiabetic drug, demonstrated potential anticancer effects via modulation of these interconnected pathways. Overall, this review underscores a biologically intertwined relationship between diabetes mellitus and cancer, supporting the development of integrated therapeutic strategies targeting shared metabolic and signaling mechanisms.

## 1. Introduction

Diabetes mellitus (DM) is a multifactorial metabolic disease characterized by chronic hyperglycemia caused by abnormalities in insulin secretion, insulin sensitivity, or the simultaneous impairment of both processes. It is one of the major global public health concerns that affects millions of people worldwide and contributes to high rates of morbidity and mortality [1]. The most recent International Diabetes Federation (IDF) and Diabetes Atlas (2026) estimate that diabetes affects 11.1% of adults aged 20–79 years worldwide, equivalent to approximately one in nine individuals, with more than 40% of cases remaining undiagnosed. Globally, 589 million adults were estimated to be living with diabetes in 2024, while IDF projections suggest that this figure will increase to approximately 853 million by 2050, corresponding to about one in eight adults and a projected 46% rise in disease burden [2].

Type 2 diabetes mellitus (T2DM) results from a complex interaction of genetic, epigenetic, and environmental factors. Progressive insulin resistance and pancreatic β-cell dysfunction mainly contribute to this disease. Chronic β-cell dysfunction reduces insulin synthesis, thereby reducing insulin secretion. In parallel, insulin resistance is characterized by a diminished cellular response to insulin, leading to impaired lipoprotein lipase activity and dysregulated lipid metabolism, which in turn elevates circulating free fatty acid levels and contributes to the development of type 2 diabetes mellitus [3]. The molecular mechanisms governing insulin synthesis, secretion, and tissue responsiveness are tightly regulated, as insulin action must precisely match metabolic demands. Disruption of these tightly controlled processes leads to metabolic imbalance, which underlies the pathophysiology of T2DM [4].

Cancer is a complex condition characterized by unregulated proliferation of abnormal cells with the ability to invade and metastasize distant organs. This proliferation and invasion is driven by the activation of oncogenes and/or the inactivation of tumor suppressor genes that collectively promote dysregulated cell cycle progression and evasion of programmed cell death (apoptosis) [5]. Cancer remains one of the major global health challenges, affecting nearly one in five individuals during their lifetime. As a leading public health concern of the 21st century, it accounted for an estimated 20 million newly diagnosed cases and 9.7 million deaths worldwide in 2022. Lung cancer alone contributed approximately 2.5 million new cases, representing 12.4% of the global cancer burden [6]. At the molecular level, cancer development is further driven by widespread alterations in multiple signaling pathways, reflecting the highly complex nature of intracellular signal transduction. These signaling cascades entail three-dimensional protein interactions that take place in specific cellular regions; alterations of these interactions may trigger cancer pathogenesis and development [7]. Also, metabolic reprogramming enables cancer cells to alter their metabolism of glucose, lipids, and amino acids to meet their unique growth requirements. Each nutrient’s source and metabolic pathway are part of the complex and varied spectrum of tumor cellular metabolism [8].

The relationship between diabetes and cancer is biologically complex. Diabetes has been implicated in cancer initiation, progression, and metastatic potential through metabolic alterations that dysregulate cancer-associated signaling pathways and promote epithelial-to-mesenchymal transition (EMT), largely driven by hyperglycemia and hyperinsulinemia [9]. In diabetic patients, elevated glucose levels lead to increased insulin release from pancreatic β-cells. This elevated insulin can directly or indirectly affect the Insulin-Like Growth Factor 1 Receptor (IGF-1R). When insulin activates IGF-1R, it triggers several signaling pathways, including PI3K/AKT, MAPK, JAK/STAT, mTOR, Src (Sarcoma), and focal adhesion kinase (FAK). These pathways collectively promote cancer cell proliferation, survival, and migration [10,11,12]. Furthermore, high blood glucose levels increase the production of reactive oxygen species (ROS) and pro-inflammatory cytokines, such as interleukin-6 (IL-6) and tumor necrosis factor-alpha (TNF-α), compared with individuals without diabetes [13]. Increased production of ROS, IL-6, and TNF-α may facilitate tumor growth by promoting oxidative stress and chronic inflammation. Despite sharing several risk factors, type 2 diabetes and cancer have limited evidence of a common etiology. Rather, the increased cancer risk is thought to arise primarily from diabetes-associated metabolic disturbances, such as elevated fasting insulin concentrations and excess adiposity [14]. Even though several type 2 diabetes susceptibility genes, for example, Type 2 diabetes risk alleles in the *TCF7L2* gene, have been found to elevate the risk of colorectal cancer [15], while PPARG (encoding peroxisome proliferator-activated receptor γ (*PPARγ*)) lowers the risk of prostate cancer pathogenesis [16]. Furthermore, reducing cancer incidence and increasing survival of cancer patients, followed by therapeutic intervention of antidiabetic medication (Metformin), imply a complex interplay between the etiopathology of both diseases [17].

Studies are going on to explore the metabolic connections between diabetes and cancer, aiming to identify shared therapeutic targets to address the comorbidity of cancer and diabetes in clinical practice. However, there is still very little understanding of the shared etiology and causality between these two heterogeneous diseases. Thus, the purpose of this systematic review is to explore the associated genetic and metabolic pathway alterations in patients with diabetes and cancer. Also discussing the potential of repurposing antidiabetic drugs as a cancer preventive agent.

## 2. Materials and Methods

### 2.1. Search Strategy

This investigation was conducted in accordance with the Preferred Reporting Items for Systematic Reviews and Meta-Analyses (PRISMA) 2020 reporting standard [18]. Internet databases, including Google Scholar, and PubMed, were searched thoroughly for relevant literature. Articles published between January 2015 and September 2026 that discussed the connection between diabetes mellitus and cancer were included in this search.

For PubMed, the search strategy used the following Boolean search string:

(Cancer[Title/Abstract] OR Neoplasm[Title/Abstract]) AND (Diabetes[Title/Abstract] OR Diabetes Mellitus[Title/Abstract]) AND (Signaling[Title/Abstract] OR “Cell Signaling”[Title/Abstract] OR “Signal Transduction”[Title/Abstract]) NOT (Review[Publication Type] OR Systematic Review[Publication Type]).

For Google Scholar, the search was conducted using the keywords “diabetes mellitus,” “cancer,” “cell signaling pathway,” and “metabolic alteration.” These terms were combined to identify studies addressing the relationship between diabetes mellitus and cancer, with particular emphasis on metabolic alterations and cell-signaling mechanisms.

**Figure 1:**
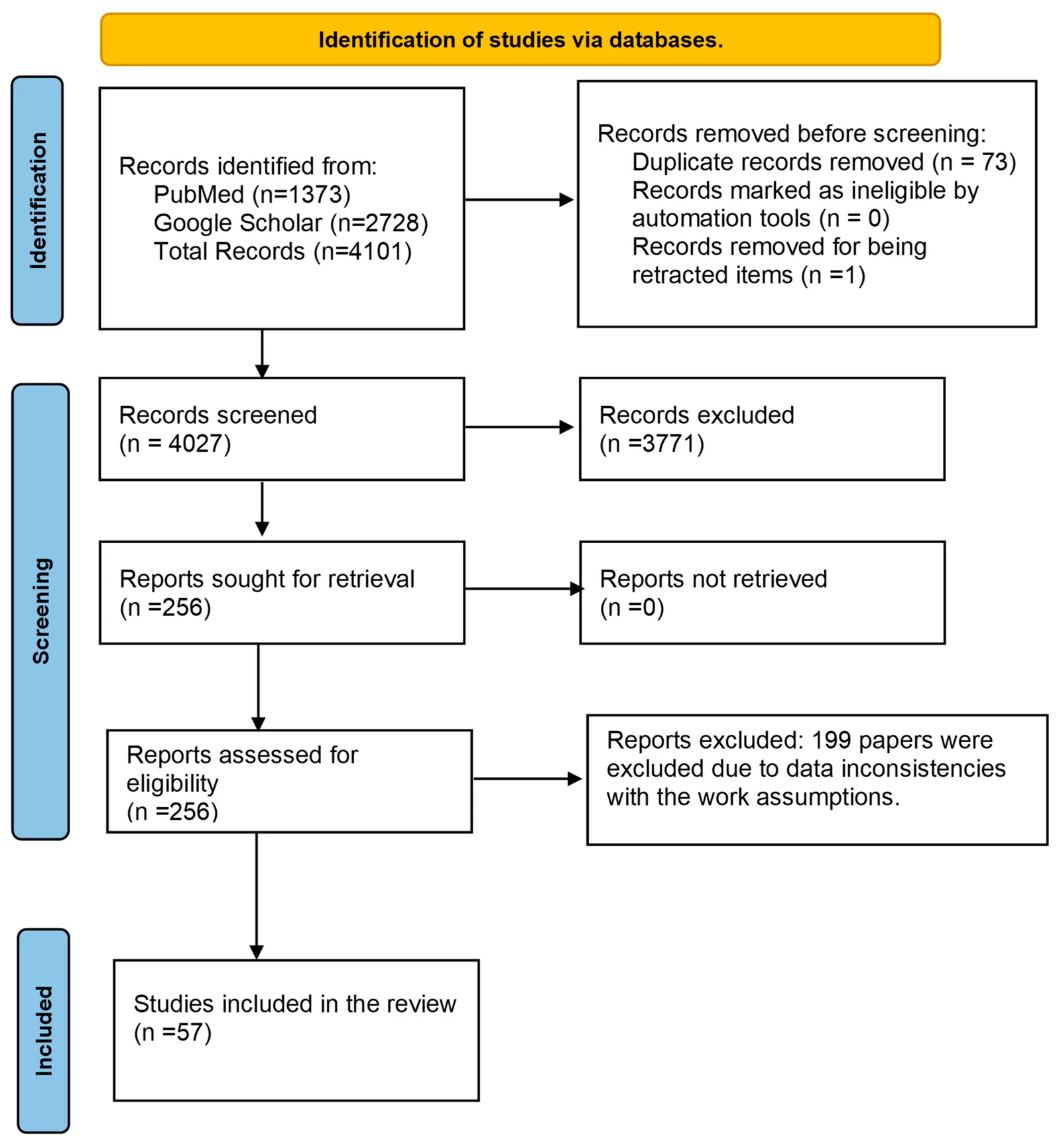
The PRISMA flow diagram visually summarizes this selection process, highlighting the steps from initial identification to final inclusion.

The search focused on literature investigating the relationship between diabetes mellitus and cancer, including epidemiological associations, metabolic alterations, molecular mechanisms, cell-signaling pathways, and potential therapeutic implications. Two reviewers separately screened the abstract and title. A third reviewer assessed any disputes about inclusion. A PRISMA flow chart was used to demonstrate the literature search procedure (Figure 1). To make sure that no relevant research was missed, the references and associated meta-analyses of the chosen papers were also retrieved and examined from other databases such as Scopus, ScienceDirect, and SpringerLink.

### 2.2. Inclusion Criteria

The inclusion criteria for study selection were designed using the PECOS framework, as summarized in Table 1. The population comprised individuals or experimental biological systems relevant to the investigation of diabetes mellitus and cancer. Diabetes mellitus was considered the principal exposure or metabolic condition of interest, while non-diabetic populations or appropriate experimental controls were considered comparators where applicable. Eligible outcomes included cancer incidence and risk as well as cancer-related clinical or prognostic outcomes, metabolic alterations, molecular interactions, signaling pathways, cellular responses, and other mechanistic evidence relevant to the biological crosstalk between diabetes mellitus and cancer.

Original research articles published between January 2015, and September 2026 were eligible if they investigated the relationship between diabetes mellitus and cancer or examined biological mechanisms linking the two conditions. Studies addressing epidemiological associations, molecular and cellular mechanisms, metabolic alterations, prognostic implications, and therapeutic or drug-repurposing perspectives were considered. Preclinical studies, including animal and in vitro investigations, were eligible when they provided mechanistic evidence relevant to the diabetes-cancer relationship. Studies investigating metformin in the context of diabetes–cancer interactions, including its potential effects on metabolic, molecular, cellular, or signaling pathways, were also considered when relevant to the objectives of the review.

### 2.3. Exclusion Criteria

Review articles, meta-analyses, books, book chapters, conference abstracts, editorials, commentaries, and case reports, were excluded from this review. In addition, studies published before 2015, non-English publications, studies focusing solely on either diabetes mellitus or cancer, studies lacking full-text availability or sufficient data, and studies that did not evaluate the association between diabetes mellitus and cancer were also excluded.

### 2.4. Study Selection

Two reviewers independently performed a two-stage screening approach to select the studies. Initially, titles and abstracts were assessed against predefined eligibility criteria, followed by full-text evaluation of all potentially relevant studies. Any discrepancies between the reviewers were resolved through discussion, and when necessary, a third reviewer was consulted to reach a consensus.

The database search yielded 4101 records. After excluding 73 duplicate records and one retracted publication, 4027 articles were retained for screening. Following the initial title and abstract assessment, 3771 records were excluded. The remaining 256 articles underwent full-text evaluation, resulting in the exclusion of 199 studies that did not satisfy the eligibility criteria. Finally, 57 studies met the inclusion criteria and were incorporated into the qualitative synthesis. The complete study selection process is illustrated in the PRISMA flow diagram (Figure 1).

### 2.5. Data Extraction

Data extraction was performed independently by two reviewers using a standardized data extraction form, and a third reviewer subsequently verified the extracted data. The data extraction sheet included the article name, the authors’ names, the publication year, the study type, and the matching criteria. The outcomes of interest were the cell metabolic changes, cell signaling, molecular interactions, and overall crosstalk between cancer and diabetes mellitus.

### 2.6. Risk of Bias Assessment

SYRCLE (animal studies), in vitro checklist (cell line studies), ROBINS-E (human observational studies), RoB 2 (clinical trial), tools were used for the assessment of risk of bias in this observational study (Supplementary Information, Table S1).

### 2.7. Data Synthesis

Data from the included studies were synthesized narratively due to heterogeneity in study designs, cancer types, and reported outcomes. Findings were organized according to cancer type, diabetes status, and metformin-related therapeutic effects. Key results, including molecular mechanisms and risk associations, were compared and summarized across studies. No meta-analysis was performed due to variability in study characteristics.

## 3. Results

### 3.1. Summary of Included Studies

A total of 57 studies were chosen based on the PRISMA guidelines. Table 2 summarizes the features of the included studies, such as examining the relationship between cancer and diabetes mellitus. It includes the authors, country of origin, type of cancer, related factors, and the importance of the results.

### 3.2. Pancreatic Cancer

Several studies have investigated the association between diabetes mellitus (DM) and pancreatic cancer and identified multiple molecular mechanisms underlying this relationship. For example, hyperglycemia has been reported to promote the initiation and progression of pancreatic cancer by activating the Wnt/β-catenin signaling pathway and to accelerate the formation of precancerous pancreatic lesions [50]. Additionally, the AKT/mTORC signaling pathway has been implicated in pancreatic cancer progression in patients with diabetes [51]. High-glucose conditions promote pancreatic cancer development by enhancing epithelial–mesenchymal transition (EMT) and cancer stem cell (CSC)-associated phenotypes. In H6c7-Kras cells, hyperglycemia activates TGF-β1 signaling through increased TGF-β1 secretion, resulting in downregulation of E-cadherin, upregulation of Nestin, and increased meroclone formation [52]. A reduction in E-cadherin expression was also detected in the pancreatic ducts of hyperglycemic mice, whereas no such decrease was observed in normoglycemic control animals [52].

Also, several studies have reported on the bidirectional and complex relationship between diabetes and pancreatic ductal adenocarcinoma. For instance, Pang W et al. explored that the exosomal miR-19a secreted by pancreatic cancer cells impaired glucose-stimulated insulin secretion (GSIS) in pancreatic β cells, suggesting a role in β-cell dysfunction [21]. Similarly, Parajuli et al. demonstrated a significant and selective loss of β-cells during the early preneoplastic stage in a KrasG12D-driven pancreatic ductal adenocarcinoma mouse model. Increased TGF-β signaling was associated with β-cell apoptosis and reduced β-cell mass; these in turn induce new onset of diabetes [53]. Epidemiological evidence suggests that both newly diagnosed type 2 diabetes and increasing glycated hemoglobin (HbA1c) levels are independently associated with a higher risk of pancreatic cancer. The bidirectional relationship between diabetes and cancer is also evident from treatment-related metabolic disturbances. Capivasertib, an AKT inhibitor used in breast cancer therapy, can disrupt insulin signaling and induce severe hyperglycemia. The two diseases appear to share common pathogenic mechanisms, as reflected by the efficacy of certain therapeutic agents in both conditions. Notably, protein tyrosine phosphatase 1B (PTP1B), a well-established therapeutic target for diabetes and obesity, has also been identified as a potential target for pancreatic cancer by regulating the PKM2/AMPK/mTORC1 signaling pathway [19]. Another molecule of interest is S100A6 (calcyclin), a calcium-binding protein that regulates intracellular calcium signaling. Evidence suggests that S100A6 is associated with type 2 diabetes, while its overexpression in pancreatic cancer has been correlated with reduced overall survival, highlighting its potential role in disease progression [20]. Moreover, insulin therapy exhibited a stronger association with pancreatic cancer initiation, while metformin showed a comparatively weaker association relative to no medication use [22]. This indicates the potential role of metformin as an anticancer therapeutic in patients with pancreatic cancer.

### 3.3. Breast Cancer

Association between T2DM and breast cancer through metabolic, hormonal, inflammatory, and genetic mechanisms has been evident in several studies. Chronic hyperinsulinemia was shown to activate the PI3K/AKT pathway in breast cancer cells, promoting proliferation in hormone-responsive MCF-7 cells and enhancing migratory behavior in triple-negative MDA-MB-231 cells, whereas hyperglycemia alone had limited effects on these phenotypes [54]. Hyperglycemia has been shown to increase mesenteric estrogen-dependent adipogenesis (MEDAG) expression, resulting in inhibition of AMPK activity and autophagy, thereby facilitating breast cancer progression [23]. Metabolic alterations associated with diabetes have also been implicated in breast cancer progression. For example, glucose starvation, combined with metformin treatment, was found to activate the unfolded protein response (UPR) in the endoplasmic reticulum via the ATF4/ATF3/CHOP signaling pathway, leading to heightened cellular stress and subsequent cancer cell death [76]. In a randomized placebo-controlled trial among postmenopausal breast cancer survivors with overweight or obesity, metformin treatment was associated with alterations in breast-tissue gene expression, immune-response pathways, amino-acid metabolism, and steroid hormone biosynthesis, suggesting potential molecular mechanisms underlying its chemopreventive effects [55]. Obesity-induced metabolic alterations, especially the release of adipocyte-derived factors, contribute to increased proliferation and motility of breast cancer cells [24]. Resistin, a bioactive adipokine released from 3T3-L1 adipocytes, together with its receptor CAP1, is markedly upregulated in breast cancer cells and primary breast tumors. Increased expression of the resistin–CAP1 axis has been linked to unfavorable clinicopathological features and poorer patient outcomes, including shorter overall and relapse-free survival [24]. Abnormal IGF signaling represents another molecular alteration observed in breast cancer. Reduced expression of IGFBP6, an important regulator that suppresses IGF-II-mediated tumor growth, has been reported in breast cancer patients with diabetes mellitus relative to those without diabetes, suggesting impaired regulation of the IGF axis in diabetic breast cancer [25]. Evidence suggests that RAGE also contributes to breast cancer progression by modulating insulin and IGF signaling. Inhibition or genetic ablation of RAGE attenuated the activation of IR and IGF-1R, which in turn impaired breast cancer cell proliferation, migration, and the formation of patient-derived mammospheres [26].

Inflammatory pathways have also been associated with T2DM and breast cancer patients. For instance, use of insulin in patients with T2DM was associated with elevated leptin, C-reactive protein (CRP), tumor necrosis factor-alpha (TNF-α), and lower C-peptide concentrations, and these factors were linked to poorer breast cancer outcomes [56]. In addition, GM-CSF and IL-5 were reported to drive breast cancer metastasis to the lungs in obese mouse models by increasing neutrophil accumulation in pulmonary tissue [27]. Altered microRNA expression has also been observed in breast cancer patients with diabetes mellitus. Compared with non-diabetic patients, those with diabetes exhibit significantly lower expression of miR-145 [28]. In contrast, miR-29a was upregulated in diabetic breast cancer patients, and silencing this microRNA inhibited cancer cell proliferation, migration, and invasion [30]. Additionally, the expression of miR-124a and miR-30d has been associated with insulin resistance and may contribute to breast cancer development in patients with diabetes mellitus [29]. Therefore, the etiopathology of breast cancer and DM share several signaling networks and targeting the common signaling pathways could provide better therapeutic outcomes for patients.

### 3.4. Prostate Cancer

Emerging evidence indicates that type 2 diabetes mellitus (T2DM) influences the development and progression of prostate cancer through multiple metabolic and molecular mechanisms. For example, increased expression of insulin-like growth factor 1 receptor (IGF-1R) was positively correlated with tumor content exclusively in prostate cancer patients with diabetes, suggesting a role for IGF signaling in diabetes-associated tumor progression [57]. In addition, a computational study investigating molecular associations between type 2 diabetes and prostate cancer identified six overlapping genes and five overlapping modules, indicating potential common pathogenic pathways [58]. Clinical studies suggest that comorbid diabetes mellitus adversely affects the prognosis of prostate cancer, resulting in reduced survival of affected patients [31]. It is also reported that the expression levels of androgen receptor (AR) were correlated with tumor content solely in patients with diabetes. Enhanced Androgen signaling was heightened in the prostate cancer tissues from men with T2DM, and AR expression was positively correlated with prostate-specific antigen (PSA, KLK3), prostate-specific membrane antigen (PSMA, FOLH1), and the cell proliferation marker Ki-67 [31]. Furthermore, Qi et al. reported that although patients with T2DM exhibit a higher overall cancer incidence compared to the general population, long-term use of certain antidiabetic agents, such as glipizide, was associated with a reduced cancer risk [71].

### 3.5. Colorectal Cancer

T2DM and colorectal cancer (CRC) shared several signaling pathways in disease pathogenesis and progression. For example, Teng et al. demonstrated that insulin and IGF-1, diabetic regulators, promote the phosphorylation of extracellular signal-regulated kinase 1/2 (ERK1/2) and c-Jun N-terminal kinase (JNK), thereby activating these pro-tumorigenic signaling pathways of colon cancer cells (MC38) [32]. Inhibition of ERK1/2 and JNK using PD98059 (a MEK inhibitor) and SP600125 (a JNK1 inhibitor), respectively, significantly suppresse the anti-apoptotic protein Bcl-2, upregulating the pro-apoptotic protein Bax expression, promoting apoptosis, and suppressing MC38’s cellular proliferation [32]. Also, increased growth of in vivo tumors in T2DM animals with higher endogenous insulin and IGF-1 supports the link between T2DM and CRC pathogenesis [32]. Diabetes mellitus also induced endothelial-mesenchymal transition (EndMT), thereby promoting CRC progression [59]. Diabetes mellitus is associated with loss of Platelet Endothelial Cell Adhesion Molecule-1 (PECAM-1) from the endothelial membrane, leading to its accumulation near the nucleus. This accumulation promotes the entry of nuclear β-catenin, which drives EndMT and contributes to the progression of CRC [59]. Upregulation of the 14-3-3 zeta protein has been observed in colorectal cancer tissues from diabetic patients. Experimental suppression of this protein reduced colorectal cancer cell proliferation and induced apoptosis, suggesting a potential role in diabetes-associated CRC progression [33]. Evidence suggests, PTPRN (receptor-type tyrosine-protein phosphatase-like N) autoantibodies may serve as a diagnostic marker to differentiate patients with coexisting T2DM and colorectal cancer from those with diabetes alone. Moreover, high PTPRN expression was found to predict an unfavorable prognosis, as reflected by decreased survival among CRC patients [34]. Another shared metabolite, α-hydroxybutyrate (α-HB), was found to promote sustained nuclear retention of NF-κB, increase TNF-α production, and impair insulin secretion, which, in turn, accelerates CRC progression [35]. Treatment of these CRC patients with metformin was reported to counteract these effects [35]. The AMPK/mTORC1/Nox4 signaling axis has also been implicated in the association between diabetes and CRC. Pharmacologic activation of AMPK, inhibition of mTORC1, and blockade of Nox4 can reduce reactive oxygen species (ROS) production, restore the proper signaling of 8-oxoguanine DNA glycosylase/8-oxodG, and slow the progression of CRC in a diabetic environment [60]. Furthermore, aberrant activation of the WNT/β-catenin pathway has been documented in CRC patients with concomitant diabetes mellitus [36]. Several studies have investigated the molecular basis of colon cancer in the context of T2DM. Patients with both conditions exhibited higher interleukin-2 (IL-2) levels than those with either disease alone or healthy controls [37]. Additionally, upregulation of the long non-coding RNA ANRIL and downregulation of miR-186-5p promoted colon cancer progression. Hyperglycemia further enhanced the oncogenic effects of ANRIL by increasing glucose metabolism and cell proliferation while inhibiting apoptosis through the regulation of miR-186-5p and glycolytic enzymes [38]. Epidemiological evidence from the Global Burden of Disease-2023 analysis identified elevated fasting blood glucose as an important metabolic risk factor contributing to the burden of early-onset colorectal cancer (EOCRC) in BRICS countries. Furthermore, in a retrospective cohort of 236 patients with colorectal cancer in China, diabetes was independently associated with poorer overall survival (HR = 4.35, 95% CI: 1.59–11.93), supporting a potential link between metabolic dysfunction and adverse CRC outcomes [61].

### 3.6. Other Cancers

Diabetes mellitus has also been linked to the onset and spread of some different cancers, such as oral, lung, and gastric cancers. Hyperglycemia in gastric adenocarcinoma raises the expression of matrix metalloproteinases (MMPs), especially MMP-9, which triggers the MAPK signaling pathway and advances tumor growth by forming AP-1 heterodimers [62]. In non-small cell lung cancer (NSCLC), miR-1231 expression was significantly associated with clinicopathological characteristics in diabetic patients and has been identified as an independent prognostic biomarker [40]. Furthermore, expression of the proliferation marker Ki-67 was significantly elevated in patients with lung adenocarcinoma in situ (AIS) and concomitant T2DM compared to those with AIS alone [43]. Conversely, among NSCLC patients with pre-existing diabetes, SGLT2 inhibitor, a new class of antidiabetic drug, use was associated with lower mortality, suggesting that antidiabetic treatment may also influence cancer outcomes [63]. Expression levels of PGC1-α and PGC1-β were significantly elevated in oral squamous cell carcinoma compared with healthy controls, indicating a potential role in tumor metabolism. However, OSCC patients with diabetes exhibited modestly lower expression of these genes than non-diabetic OSCC patients, suggesting that diabetes may influence the metabolic regulation of oral cancer [41]. Furthermore, salivary TNF-α levels differed significantly between patients with oral squamous cell carcinoma (OSCC) with and without diabetes, supporting its potential involvement in OSCC pathogenesis [42]. Collectively, these findings suggest that diabetes-associated molecular alterations may contribute to the pathogenesis and progression of diverse malignancies beyond the major cancer types discussed above.

### 3.7. Metformin: A Therapeutic and Mechanistic Link Between Diabetes and Cancer

Several studies have investigated the anticancer effects of metformin, a clinically used antidiabetic drug, in different cancer types. For example, in melanoma cells, metformin administration elevates mitochondrial Reactive Oxygen Species (mtROS) and cell-surface glucose transporter (Glut)-1 expression, resulting in enhanced IFN-γ production by CD8+ tumor-infiltrating T lymphocytes (CD8 TILs) [44]. Enhanced CD8 TIL proliferation and IFN-γ secretion were associated with decreased glycolysis and oxidative phosphorylation in tumor cells, in synergy with the effect of anti-PD-1 therapy [44]. A phase II clinical trial involving individuals with oral premalignant lesions demonstrated that metformin induces favorable histological responses and modulates the mTOR signaling pathway [64]. Metformin has also been shown to inhibit ovarian cancer-related pathways through multiple mechanisms. Hart et al. [45] demonstrated that metformin reduced the expression of succinyl-CoA ligase (SUCLG2), a tricarboxylic acid (TCA) cycle enzyme, thereby activating prolyl hydroxylases (PHDs) and promoting the degradation of hypoxia-inducible factor-1α (HIF-1α) in mesothelial cells. In a separate study, Hart, Chiyoda, et al. [46] reported that metformin suppressed hypoxia-induced sphingosine kinase 1 (SPHK1) expression and its downstream sphingosine-1-phosphate (S1P) signaling pathway, both of which are implicated in ovarian cancer progression. In obese, non-diabetic women with early endometrial cancer, metformin treatment increased progesterone receptor and PTEN expression while decreasing estrogen receptor expression [47]. In-vitro experiments also have showed reduced cancer cell numbers and altered expression of steroid receptor–related genes [47]. In human osteosarcoma cells, metformin induced G2/M phase cell-cycle arrest and promoted programmed cell death and autophagy through activation of the ROS/JNK signaling pathway [65]. Metformin also impaired cancer stem-like cell (CSC) properties in osteosarcoma by inhibiting sphere formation and reducing the expression of stemness-associated transcription factors Oct-4 and Nanog [66]. In the breast cancer model, it suppresses estrogen receptor (ER)-mediated signaling by reducing E2-inducible estrogen response element (ERE) activity and downregulating ERα target genes, leading to decreased proliferation [48]. In addition, metformin blocks lithocholic acid-stimulated ROS production, suppresses NF-κB signaling, which in turn blocks IL-8 upregulation, and thereby prevents IL-8-mediated endothelial cell proliferation and tubelike formation [67]. Furthermore, the drug reduced IL-6 upregulation and inhibited epithelial–mesenchymal transition, cell motility, and invasion [49]. In CRC associated with diabetes mellitus, metformin was reported to modulate the Notch1/Hes1 signaling, which was associated with abnormal cellular proliferation and differentiation [68]. Metformin reversed insulin-induced oxaliplatin resistance in human colon cancer by activating the AMPK/Erk signaling pathway [69]. A novel rhodium(III) hybrid metallodrug combining metformin with an oxoaporphine alkaloid (Rh-Met) was also shown to suppress the PI3K/AKT/mTOR pathway and modulate glucose metabolism in a T2DM–bladder cancer model. In a T2DM–tumor model, Rh-Met simultaneously inhibited tumor progression and reduced hyperglycemia, highlighting its potential as a dual therapeutic strategy for managing diabetes and cancer [70]. Likewise, the antidiabetic drug glipizide exhibited anticancer activity, whereas the anticancer drug capmatinib showed potential to improve insulin resistance and T2DM [71,72]. A randomized trial in 272 non-diabetic patients found that one year of metformin significantly reduced colorectal adenoma recurrence after polypectomy, without significant changes in blood glucose or lipid parameters, suggesting a potential glucose-independent chemopreventive effect of metformin [77]. Together, these findings highlight the substantial overlap in the molecular signaling pathways between T2DM and cancer, supporting opportunities for drug repurposing to improve the management of both diseases.

## 4. Discussion

**Figure 2:**
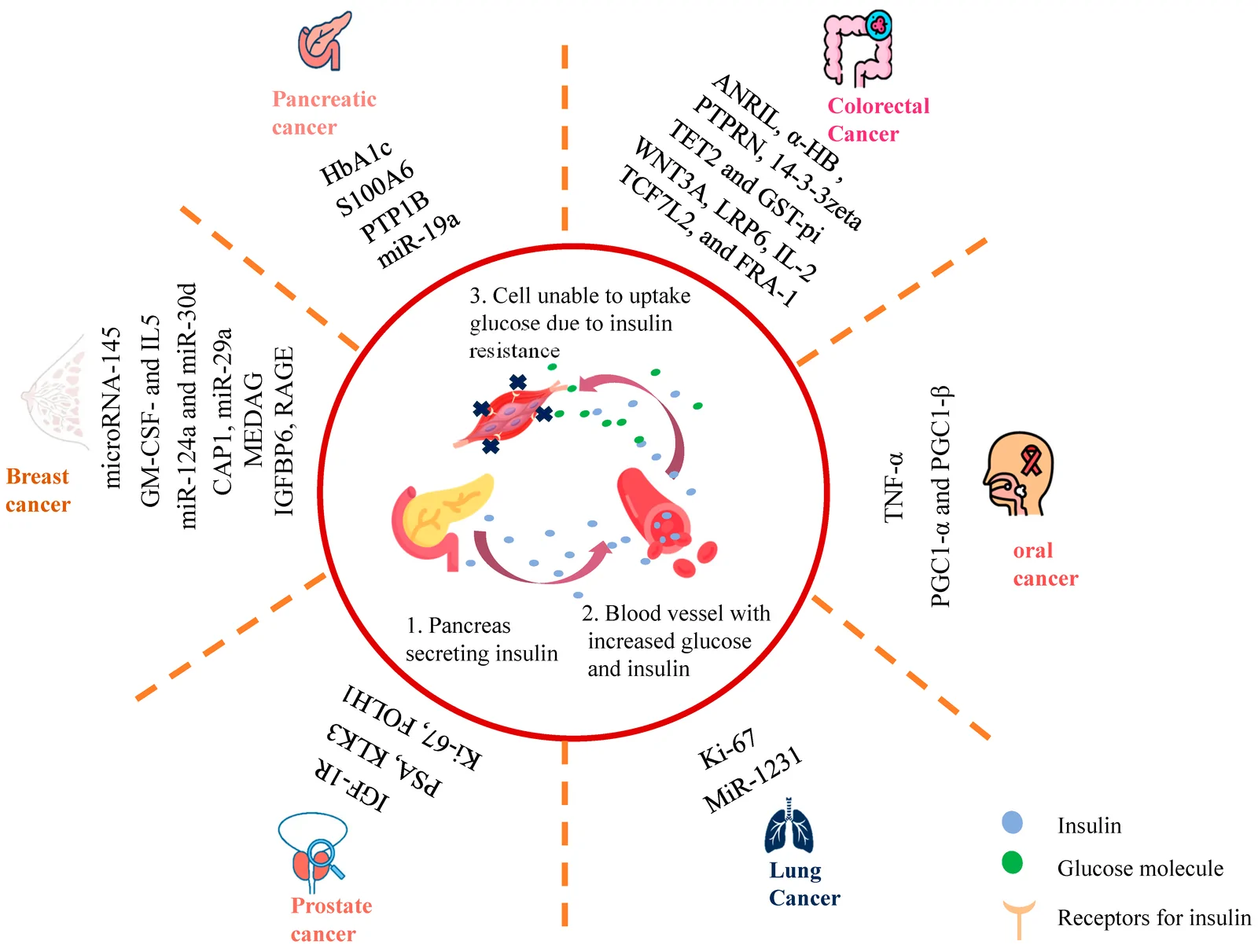
**Representation of genes associated with cancer and diabetes.** Numerous genes have been shown to have potential biomarkers or common linkages; however, their functions can be complicated and occasionally unique to cancer.

Diabetes mellitus and cancer are among the most prevalent chronic diseases worldwide and are increasingly recognized as biologically interconnected conditions that impose a substantial burden on global health. Although the precise nature of this relationship remains incompletely understood, growing evidence indicates that dysregulated metabolic pathways, chronic inflammatory responses, and genetic mechanisms may facilitate the shared pathogenesis and progression of both diseases. The findings of this systematic review offer proof of a robust correlation between diabetes mellitus (DM) and a higher risk of several forms of cancer. It is suggested that a wide range of biological mechanisms, including hyperglycemia, hyperinsulinemia, insulin resistance, chronic inflammation, oxidative stress, and genetic alterations, may contribute to cancer initiation and progression in individuals with diabetes. These observations are consistent with previous epidemiological studies demonstrating a higher incidence of cancer among patients with DM. Furthermore, emerging genetic evidence suggests that common molecular pathways and susceptibility genes may underline the complex interplay between diabetes and cancer (Figure 2). A deeper understanding of these shared biological mechanisms can pave the way to the development of more effective preventive strategies, like enhancing early cancer detection, and facilitating the invention of novel therapeutic targets for both diseases.

There is a complicated, reciprocal association between pancreatic cancer and type 2 diabetes mellitus (T2DM); about half of individuals with pancreatic cancer also have T2DM or reduced glucose tolerance. New-onset diabetes (NOD) may be an early sign of pancreatic cancer and may offer a chance for its early detection, even though long-term type 2 diabetes is a known risk factor for the disease [78]. This connection has been explained by a number of biological mechanisms. Chronic hyperglycemia, compensatory hyperinsulinemia, and insulin resistance typically precede diabetes and may contribute to the development of cancer. An increased risk of pancreatic cancer has been associated with elevated levels of insulin, C-peptide, and glycated hemoglobin (HbA1c) [22]. In addition, accumulating evidence indicates that hyperglycemia promotes pancreatic tumor initiation and progression by activating the Wnt/β-catenin signaling pathway and may facilitate the development of precancerous pancreatic lesions [50]. The development of pancreatic cancer in diabetic people has also been found to be linked to dysregulation of the AKT/mTOR pathway [51]. Hyperglycemia may also enhance EMT and CSC-like properties through activation of TGF-β signaling. High glucose conditions have been shown to increase TGF-β1 secretion, reduce E-cadherin expression, and promote the acquisition of mesenchymal characteristics in pancreatic duct epithelial cells, thereby facilitating tumor development [52]. Furthermore, shared molecular pathways between diabetes and pancreatic cancer have been identified. Protein-tyrosine phosphatase 1B (PTP1B), a validated therapeutic target in diabetes and obesity, has also emerged as a potential target in pancreatic cancer, where its inhibition suppresses tumor progression through modulation of the PKM2/AMPK/mTORC1 signaling axis [19]. At the genetic level, Weighted Gene Co-expression Network Analysis (WGCNA) identified common molecular signatures between pancreatic cancer and T2DM. Functional enrichment analysis revealed significant involvement of genes (*n* = 44) regulating endodermal cell fate specification. Among the 44 shared genes, S100A6 was identified as a key hub gene, a potential immune-related biomarker, and a candidate for therapeutic target. Its high expression in pancreatic cancer and association with poorer overall survival further support the existence of shared molecular mechanisms linking T2DM and pancreatic cancer [20]. As we have already seen, the relationship between pancreatic cancer and diabetes is bidirectional. Pancreatic cancer-derived exosomal miR-19a has been shown to impair glucose-stimulated insulin secretion in β-cells, while increased TGF-β signaling may promote β-cell apoptosis and loss of β-cell mass. These findings provide a potential explanation for the development of new-onset diabetes in patients with pancreatic cancer [21,53]. Overall, accumulating evidence supports the concept that metabolic dysfunction, aberrant molecular signaling, and shared genetic factors collectively drive the intertwined pathology of T2DM and pancreatic cancer. A deeper understanding of these interconnected mechanisms could facilitate the identification of reliable biomarkers and the development of targeted therapeutic strategies for the early detection and effective management of pancreatic cancer in patients with diabetes.

The most common disease in women, breast cancer, is linked to type 2 diabetes mellitus (T2DM), according to an increasing amount of epidemiological data. According to a meta-analysis of 5 case–control and 15 cohort studies, diabetes is linked to a roughly 20% higher incidence of breast cancer when compared to the non-diabetic population [73]. This association suggests that metabolic dysregulation plays an important role in breast carcinogenesis. Obesity-related metabolic alterations and adipocyte-derived factors are key contributors to breast cancer progression. Adipocytes within the tumor microenvironment promote cellular reprogramming, enhancing proliferation and the acquisition of more motile phenotypes in both ER-positive and triple-negative breast cancer cells. Among adipokines, resistin has been identified as an important mediator linking obesity, insulin resistance, and breast cancer [24]. Resistin secreted by adipokines such as 3T3-L1 cells exerts its effects through the CAP1 receptor, which is aberrantly expressed in breast cancer cells and is linked to aggressive tumor features and poor prognosis [24]. There may be a common molecular axis between obesity, diabetes, and breast cancer since resistin-CAP1 signaling can trigger inflammatory pathways that lead to metabolic dysfunction and insulin resistance. Additionally, by directly boosting the growth of breast tissue and indirectly by raising the levels of IGFs, estrogens, and other growth-promoting hormones in the blood, hyperinsulinemia and insulin resistance further contribute to the development of breast cancer. These hormonal and metabolic changes produce a tumor-promoting milieu that can accelerate the onset and spread of breast cancer [79]. In addition, T2DM may negatively influence breast cancer screening and treatment outcomes due to overlapping metabolic and clinical complications [80]. Furthermore, inflammatory signaling pathways also play a central role in the diabetes–breast cancer association. Elevated levels of inflammatory mediators such as leptin, CRP, and TNF-α have been reported in diabetic patients and are linked to poorer breast cancer outcomes. These findings highlight chronic low-grade inflammation as a key biological link between metabolic disease and tumor progression. At the molecular level, the dysregulation of miRNAs represents an additional shared mechanism between T2DM and breast cancer. For instance, people with T2DM who have breast cancer have considerably lower levels of miR-145 expression, and this downregulation is linked to unfavorable clinicopathological characteristics such as tumor size, stage, lymph node metastasis, and receptor status [28]. Restoration of miR-145 expression has been shown to suppress tumor cell proliferation and angiogenesis by targeting oncogenic pathways, including N-RAS and VEGF signaling [81]. Similarly, miR-124a and miR-30d have been implicated in insulin resistance and breast cancer development, reflecting their roles in regulating both metabolic and oncogenic processes. miR-29a has also been reported to promote breast cancer cell proliferation and metastasis in diabetic conditions through modulation of SIRT1 [30]. Altogether, these miRNAs act as important molecular regulators linking metabolic dysfunction to breast cancer progression. Overall, the evidence suggests that metabolic disturbances, including insulin resistance, hyperinsulinemia, adipokine signaling, inflammatory activation, and miRNA dysregulation, collectively contribute to the increased risk and progression of breast cancer in patients with T2DM. A better understanding of these interconnected pathways may provide potential opportunities for improved risk stratification, early detection, and targeted therapeutic strategies in this population.

Several epidemiological studies have identified T2DM as a significant risk factor for CRC, with adverse effects also observed on CRC prognosis [82]. The underlying mechanisms linking these two conditions are multifactorial and involve metabolic, inflammatory, and genetic dysregulation. Hyperinsulinemia, hyperglycemia, and dysregulation of the IGF axis contribute to CRC development by activating key proliferative signaling pathways, including PI3K/AKT, MAPK, mTOR, and WNT/β-catenin [82,83]. These pathways collectively promote cellular proliferation, survival, and tumor progression. In addition, gene expression analyses have demonstrated altered regulation of key WNT/β-catenin components such as WNT3A, LRP6, TCF7L2, and FRA-1 in diabetic CRC patients, further supporting activation of this oncogenic pathway in diabetic patients [36,84]. These findings shed new light on the biology behind the link, and experimental research helps to understand the processes by which these genes link diabetes and colorectal cancer development [85]. Immune dysregulation also plays a crucial role in the diabetes–CRC association. Elevated interleukin-2 (IL-2) levels in patients with both T2DM and CRC suggest altered immune signaling. IL-2-mediated expansion of regulatory T cells may contribute to immune tolerance within the tumor microenvironment, thereby facilitating tumor progression [37,86]. Moreover, chronic inflammatory signaling associated with adipose tissue dysfunction further enhances tumor-promoting immune responses. Moreover, metabolic reprogramming under hyperglycemic conditions provides an additional tumor-supportive environment. High glucose levels may enhance the oncogenic activity of long non-coding RNA ANRIL, leading to suppression of miR-186-5p and increased expression of glycolytic enzymes, thereby promoting CRC cell proliferation and survival [38]. Furthermore, functional polymorphisms in insulin signaling genes, including *INS*, *INSR*, *IGF1R*, *IRS1*, and *IRS2*, may influence CRC susceptibility by modulating insulin-driven mitogenic signaling pathways [74]. Collectively, these findings suggest that T2DM promotes colorectal carcinogenesis through a complex interplay of metabolic stress, chronic inflammation, immune dysregulation, and activation of oncogenic signaling pathways. Although these genes and pathways do not directly initiate cancer, they create a systemic environment that favors tumor development and progression, highlighting the importance of metabolic health in colorectal cancer prevention.

The higher incidence of NSCLC is frequently associated with aging, which may partly explain its co-occurrence with other age-related conditions like DM. Emerging evidence also suggests a strong association between aberrant miRNA expression in NSCLC and DM [87]. In NSCLC patients with DM, miR-1231 expression is significantly associated with key clinical parameters and is identified as an independent prognostic biomarker. Furthermore, nomogram-based multivariate models have demonstrated improved accuracy in predicting overall survival in this patient population [40]. In addition, proliferation marker Ki-67, which reflects tumor cell growth activity, provides additional prognostic value beyond conventional lung cancer biomarkers such as carcinoembryonic antigen (CEA) and cytokeratin 19 fragment (CYFRA 21-1) in DM patients. Elevated Ki-67 expression has been observed in lung adenocarcinoma in situ (AIS) patients with concomitant T2DM compared with AIS patients without diabetes, suggesting enhanced proliferative activity in a hyperglycemic environment [43].

One of the most common head and neck malignancies worldwide, Oral Squamous Cell Carcinoma (OSCC) is marked by a poor prognosis and a significant chance of recurrence. According to new research, diabetes mellitus not only raises the risk of OSCC but also affects treatment outcomes and the course of the illness [88]. Hyperglycemia is considered a key factor linking T2DM and OSCC, as it modulates cancer cell proliferation, migration, and apoptosis, thereby promoting tumor progression. In diabetic OSCC patients, downregulation of BRIP1, E-cadherin, and cleaved caspase-3, along with upregulation of Ki67, has been reported, indicating enhanced proliferative and reduced apoptotic activity under hyperglycemic conditions [75]. Also, Fan et al. demonstrated a genetic cross-talk between T2D and OSCC, where immune-related genes such as *ABCD1*, *C1QC*, *CSE1L*, and *PSMC4* were identified as key mediators associated with disease progression and immune regulation [89]. Inflammatory signaling also plays a critical role in OSCC progression in diabetic patients. TNF-α, a key inflammatory mediator, has been implicated in both diabetes-associated inflammation and cancer progression, potentially accelerating OSCC advancement and reducing patient survival [42].

The evidence synthesized in this review strengthens the reported association between diabetes mellitus and increased cancer risk. The molecular and genetic alterations identified across the included studies provide valuable insights into the biological mechanisms linking diabetes to cancer development and progression. Nevertheless, this review has several limitations. First, potential confounding factors shared by both diabetes and cancer, including age, sex, obesity, smoking, and alcohol consumption, were not specifically addressed. Second, although numerous molecular mechanisms have been described, the limited availability of studies evaluating cancer aggressiveness makes it difficult to determine the overall cancer risk associated with diabetes and to establish the contribution of individual genes to this increased risk. Third, rather than focusing on all forms of diabetes, this study exclusively examined DM. Fourth, the pathways linked to the genetic alterations in diabetes and cancer are not fully understood in current research. Results may also be impacted by variations in geographic locations, racial or cultural backgrounds, and the degree of use of medical services. Fifth, the current study did not prospectively register in PROSPERO, OSF, or another repository. Lastly, persons with and without diabetes mellitus may utilize cancer screening services to varying degrees. These findings should be interpreted with caution. For example, a recent systematic review and meta-analysis reported an overall inverse association between diabetes and prostate cancer risk (pRR = 0.71) across most populations, although this relationship was not observed in Asian populations. Notably, their subgroup analysis revealed that diabetic individuals with a family history of prostate cancer had a significantly higher risk of developing the disease (pRR = 1.25) [90]. Nonetheless, another meta-analysis conducted by Long et al. reported that the Asian population showed an increased risk of prostate cancer in diabetic patients [91]. Although numerous studies have suggested that metformin exerts protective effects against several types of cancer, this benefit has not been consistently observed for prostate cancer [92]. Moreover, the use of antidiabetic medications with potential anticancer properties may act as a confounding factor, making it difficult to accurately estimate the independent effect of diabetes on cancer risk.

## 5. Conclusions

Accumulating evidence indicates that diabetes mellitus and cancer are biologically intertwined conditions linked by complex metabolic, genetic, and signaling mechanisms rather than merely co-occurring through shared risk factors. Key pathophysiological alterations include hyperglycemia, hyperinsulinemia, insulin resistance, chronic low-grade inflammation, and oxidative stress, which in turn actively facilitate tumor initiation, cell proliferation, and progression across diverse malignancies, including pancreatic, breast, colorectal, prostate, and lung cancers. At the molecular level, these metabolic perturbations alter crucial signaling pathways such as Wnt/β-catenin, PI3K/AKT/mTOR, and AMPK, while dysregulated microRNAs (such as miR-19a, miR-145, and miR-29a) further modulate disease severity. Notably, a bidirectional relationship is prominent in pancreatic ductal adenocarcinoma, where cancer-derived exosomal miR-19a impairs glucose-stimulated insulin secretion to induce new-onset diabetes. Furthermore, the therapeutic efficacy of antidiabetic drugs like metformin in suppressing tumor growth highlights the clinical promise of targeting these shared oncogenic and metabolic pathways. This encourages a more integrated approach to patient care and opens the door for innovative therapeutic approaches, such as repurposing antidiabetic medications like metformin for oncology.

## 6. Future Perspectives

Current understanding shifting the clinical and research paradigms from viewing diabetes mellitus and cancer as distinct diseases toward treating them as biologically interconnected conditions. Thus, the key future directions stemming from the evidence includes, but not limited to integrated clinical care and early detection using the metabolic indicators such as new-onset diabetes, elevated HbA1c levels, or circulating biomarkers (e.g., exosomal miR-19a, S100A6, or PTPRN autoantibodies), targeting shared molecular cascades by designing dual-action therapeutics focused on interconnected signaling networks, for example Wnt/β-catenin, PI3K/AKT/mTOR, AMPK, and PTP1B, etc., expanding antidiabetic drug repurposing for better management of diabetic patients with cancers.

## Figures and Tables

**Table 1:** Eligibility criteria for this study.

PECOS	Eligibility Criteria
Population (P)	Human populations and relevant cell/animal models
Exposure (E)	Diabetes mellitus, diabetes-related alterations, or metformin
Comparator (C)	Non-diabetic, control, untreated, or relevant comparator groups
Outcome (O)	Cancer risk/incidence, prognosis, metabolic or molecular changes, signaling pathways, and diabetes–cancer crosstalk
Study design (S)	Observational, clinical/prognostic, animal, and in-vitro studies

**Table 2:** Association between patients with Cancer and Diabetes Mellitus.

Authors	Country	Cancer Type	Associated Molecules	Significance
Xu et al., 2019 [19]	China	Pancreatic Cancer	Protein Tyrosine Phosphatase 1B (PTP1B)	A potential therapeutic target for diabetes and obesity. Its inhibition can block pancreatic cancer progression by targeting the PKM2/AMPK/mTORC1 pathway.
Hu et al., 2022 [20]	China	Pancreatic Cancer	S100 calcium-binding protein A6 (S100A6)	S100A6 helped to induce the PC cells’ proliferation and migration. PC patients with high S100A6 expressions had worse overall survival (OS) than those with low expression.
Pang et al., 2021 [21]	China	Pancreatic cancer	microRNA 19a (miR-19a)	miR-19a cab causes de novo diabetes in pancreatic cancer patients by disrupting glucose-stimulated insulin secretion (GSIS).
Lu et al., 2015 [22]	Sweden	Pancreatic cancer	Hemoglobin A1c (HbA1c)	The correlation between raised HbA1c levels and an increased risk of pancreatic cancer supports the idea that new-onset diabetes is linked to an increased risk of the disease, particularly in the early stages following diagnosis.
Li et al., 2022 [23]	China	Breast Cancer	Mesenteric estrogen-dependent adipogenesis (MEDAG)	In hyperglycemic conditions, an increase in (MEDAG) expression inhibits AMPK activity and autophagy, thereby facilitating tumor progression.
Rosendahl et al., 2018 [24]	Sweden	Breast Cancer	Adenylyl cyclase-associated protein 1 (CAP1)	The adipocyte secretome promotes breast cancer cell proliferation and motility, and highlights a potential role of CAP1 regarding breast cancer outcome
Lu et al., 2024 [25]	China	Breast Cancer	Insulin-Like Growth Factor Binding Protein 6 (IGFBP6)	Typically, it is upregulated in BC, which correlates with a better prognosis, improved overall survival, and increased sensitivity to chemotherapy drugs. But in BC with T2DM, it starts to downregulate.
Muoio et al., 2023 [26]	Italy	Breast Cancer	Receptor for advanced glycation end products (RAGE)	It is pharmacological inhibition or genetic knockout halts the activation of both IR and insulin-like growth factor 1 receptor (IGF-1R), which in turn reduces cell proliferation, migration, and patient-derived mammosphere formation.
Quail et al., 2019 [27]	USA	Breast Cancer	Granulocyte-Macrophage Colony-Stimulating Factor (GM-CSF) Interleukin-5 (IL-5)	Obesity triggers chronic low-grade inflammation, disrupts tissue environment, and increases neutrophils in the lungs. More lung neutrophils boost breast cancer spread to lungs, driven by GM-CSF and IL-5.
Liu et al., 2016 [28]	China	Breast Cancer	microRNA-145	Low miR-145 expression may be a poor predictive factor for people with type 2 diabetes, and loss of miR-145 expression is linked to the development of breast cancer, worsened by the condition.
Zhang et al., 2016 [29]	China	Breast Cancer	microRNA-124a microRNA-30d	Expressions were positively correlated with BMI and glycosylated hemoglobin, as well as with insulin resistance and the development of BC in individuals with T2DM.
Li et al., 2022 [30]	China	Breast Cancer	microRNA-29a	miR-29a promoted cell growth and metastasis of BDM via targeting of SIRT1
Lutz et al., 2018 [31]	Germany	Prostate Cancer	Kallikrein-related Peptidase 3 (KLK3) Folate Hydrolase 1 (FOLH1) Ki-67	The expression levels of androgen receptor (AR) mRNA and protein are correlated with tumor content. And AR expression was positively correlated with downstream targets such as prostate-specific antigen (KLK3) and prostate-specific membrane antigen (FOLH1), as well as with the cell proliferation marker Ki-67. Suggesting enhanced tumor growth activity.
Teng et al., 2016 [32]	China	Colorectal Cancer	Extracellular-signal-regulated kinase 1/2 (ERK1/2) C-Jun N-terminal kinase (JNK)	Insulin and IGF-1 promote the phosphorylation of (ERK1/2) and (JNK). Dose-dependent treatment with ERK1/2 inhibitor PD98059 and JNK inhibitor SP600125 lead to increased apoptosis and inhibition of proliferation.
Ding et al., 2018 [33]	China	Colorectal Cancer	14-3-3zeta (isoform of the 14-3-3 protein family)	Higher expression of 14-3-3zeta was observed in colorectal cancer (CRC) tissues of diabetic patients. Knockdown of 14-3-3zeta resulted in reduced CRC proliferation and enhanced apoptosis.
Garranzo-Asensio et al., 2022 [34]	Spain	Colorectal Cancer	Receptor-type tyrosine-protein phosphatase-like N (PTPRN)	PTPRN autoantibody levels in plasma discriminate between patients with T2D, with and without CRC. High PTPRN expression correlated with decreased survival of patients with CRC.
Lv et al., 2023 [35]	China	Colorectal Cancer	α-HB (α-Hydroxybutyrate)	A common metabolite of diabetes and CRC. Impairs insulin secretion and exacerbates CRC progression and type 2 diabetes.
Wence-Chavez et al., 2017 [36]	México	Colorectal Cancer	Wnt Family Member, 3A(WNT3A), Low-Density Lipoprotein Receptor-Related Protein 6(LRP6), Transcription Factor 7 Like 2 (TCF7L2), and Fos-related antigen 1 (FRA-1)	The WNT/β-catenin pathway is upregulated in patients with CRC and DM. In CRC patients with DM, the expression levels of WNT3A, LRP6, TCF7L2, and FRA-1 were significantly higher compared to controls.
Bosek et al., 2016 [37]	Poland	Colorectal Cancer	interleukin-2	Compared to the groups without diabetes or large intestine cancer, the patients with T2DM and CC had greater levels of interleukin-2. It may be possible to link a greater level of interleukin-2 to a higher risk of CC.
Mosaad et al., 2024 [38]	Egypt	Colorectal Cancer	Antisense Noncoding RNA in the INK4 Locus (ANRIL)	Upregulation of ANRIL expression levels and a significant downregulation of miR-186-5p expression in diabetic colon cancer specimens compared to those in the non-diabetic colon cancer group were observed
Papadatos et al., 2024 [39]	Greece	Colorectal Cancer	Ten-Eleven Translocation 2 (TET2) Glutathione-S-transferase pi (GST-pi)	Patients without T2DM tended to have tumors with weak or no expression of TET2, whereas diabetic patients’ tumors showed a significantly higher percentage of strong or moderate GST-pi expression.
Li et al., 2020 [40]	China	Non-small cell lung cancer (NSCLC)	MicroRNA-1231	The mortality rate in the high miR-1231 level (≥1.775) group was 57.4%. MiR-1231 reduced the risk of cancer-related death in patients with combined NSCLC and DM. patients who underwent surgery had lower mortality rates compared to those who did not (*p* < 0.001)
Thamizhanambi et al., 2022 [41]	India	Oral squamous cell carcinoma (OSCC)	Peroxisome Proliferator-Activated Receptor Gamma Coactivator 1-Alpha (PGC1-α) Peroxisome Proliferator-Activated Receptor Gamma Coactivator 1-Beta (PGC1-β)	They did not present with a significant fold change, and the hyperglycemic status of the individual did not contribute to the expression of the target genes, as *p* values obtained for PGC1-α and PGC1-β were >0.05.
Azimudin et al., 2024 [42]	India	Oral squamous cell carcinoma. (OSCC)	Tumor Necrosis Factor alpha (TNF-α)	The TNF-α levels between the control group (51.42 ± 1.4 pg/mL), OSCC patients without DM (67.43 ± 1.7 pg/mL), and OSCC patients with DM (268 ± 8.5 pg/mL) were noted.
Chen et al., 2025 [43]	China	Lung adenocarcinoma in situ (AIS)	Ki-67	Patients with AIS exhibited a significantly higher average Ki-67 positivity rate
Nishida et al., 2021 [44]	Japan	Melanoma	CD8+ tumor-infiltrating T lymphocytes (CD8TILs) Interferon gamma (IFN-γ)	Met enhances CD8+ TIL proliferation and IFN-γ secretion, resulting in decreased glycolysis and oxidative phosphorylation in tumor cells, which promotes antitumor immunity in cancer cells.
Hart et al., 2019 [45]	USA	Ovarian Cancer	Hypoxia-inducible factor 1a (HIF1a)	Metformin triggers the breakdown of hypoxia-inducible factor 1a (HIF1a) in mesothelial cells, which disrupts tumor-stromal crosstalk.
Hart et al., 2019 [46]	USA	Ovarian Cancer	Sphingosine kinase 1 (SPHK1)	Metformin blocks hypoxia-induced sphingosine kinases (SPHK1), thereby inhibiting cancer progression.
Pabona et al., 2020 [47]	USA	Endometrial cancer	KLF transcription factor 9 (KLF9)	Metformin downregulates steroid receptor-related genes, particularly KLF9. This prevents endometrial cancer by modifying hormone signaling in endometrial cancer cells.
Kim et al., 2016 [48]	Republic of Korea	Breast Cancer	E2-inducible estrogen response element (ERE)	Metformin represses ERE luciferase activity, protein levels, and mRNA levels of E2/ERα-regulated genes, resulting in inhibition of cell proliferation
Kang et al., 2018 [49]	Republic of Korea	Colon Cancer	Interleukin-6 (IL-6)	Metformin inhibits the upregulation of IL-6 that allows epithelial–mesenchymal transition, and in turn, cell motility and invasion.
Chen et al., 2021 [50]	China	Pancreatic ductal adenocarcinoma (PADC)	Wnt/β-catenin	Diabetes mellites or hyperglycemia promotes pancreatic cancer by promoting Wnt/β-catenin signaling pathway.
Velazquez-Torres et al., 2020 [51]	USA	Pancreatic cancer	AKT/mTOR	DM2 induces metabolic changes, which in turn activate AKT/mTOR mediated pancreatic cancer pathogenesis.
Rahn et al., 2018 [52]	Germany	PADC	Transforming growth factor beta 1 (TGF-β1) and E-cadherin	DM2 promotes PADC cells to acquire cancer stem cell like phenotype by activation TGF-β1 signaling and reduced E-cadherin expression.
Parajuli et al., 2020 [53]	USA	PADC	Transforming growth factor beta (TGF-β)	Depletion of β-cells and activation of TGF-β signaling provide a link between PADC and diabetes pathogenesis.
Melloni et al., 2025 [54]	Italy	Breast cancer	PI3K/AKT and MAPK pathway	Higher insulin activates glucose metabolism and promotes breast cancer proliferation by activating PI3K/AKT-MAPK pathway.
Strømland et al., 2025 [55]	Norway	Breast cancer	CYP11A1 and CYP1B1	Breast cancer survivors treated with metformin induced changes in gene expression, resulting in prevention of new tumor growth.
Wintrob et al., 2017 [56]	USA	Breast cancer	Cytokines	Using of insulin in diabetic patients associated with elevated leptin, CRP, TNF-alfa and lower C-peptide which are associated with poor prognosis.
Broggi et al., 2021 [57]	Italy	Prostate cancer	Insulin growth factor-1 (IGF-1) receptor	Expression of IGF-1R correlated with increased prostate cancer aggressiveness in patients with diabetes.
Liu et al., 2019 [58]	China	Prostate cancer	Sarcoglycan delta (SGCD) and Rho family GTPase 3 (RND3)	Overlapping these genes negatively regulate apoptosis and mediate prostate cancer in hyperglycemic state.
Wu et al., 2023 [59]	China	Colon cancer	platelet-endothelial cell adhesion molecule 1 (PECAM-1) internalization drives β-catenin-mediated endothelial-mesenchymal transition (EndMT)	Defect or internalization of PECAM-1 activate β-catenin-mediated EndMT followed by activation of Akt/GSK-3β signaling in animal models.
Mroueh et al., 2019 [60]	Lebanon	Colorectal cancer	AMPK/mTORC1/Nox4 signaling axis	Diabetes mellitus activates AMPK/mTORC1/Nox4 signaling axis and promote colorectal cancer pathogenesis.
Zhang et al., 2026 [61]	China	Colorectal cancer	Metabolic patterns	Low fiber, and milk, high red meat and high BMI associated with diabetes mellitus and CRC onset in BRICS countries.
Chatterjee et al., 2024 [62]	India	Gastric adenocarcinoma	AP-1 transcription factor	Hyperglycemia induce MMP-9 transcription, which in turn upregulated AP-1 activity, resulting in aggregated complications in gastric cancer-hyperglycemia patients.
Luo et al., 2023 [63]	USA	Non-small cell lung cancer	Sodium-glucose cotransporter 2 (SGLT2)	Inhibition of SGLT2 associated with improved survival of NSCLC patients with pre-existing diabetes mellitus.
Gutkind et al., 2021 [64]	USA	Head and neck squamous cell carcinoma	PI3K/mTOR signaling pathway	Inhibition of mTOR activity significantly reduced oral premalignant lesions.
Li et al., 2020 [65]	China	Osteosarcoma	ROS/JNK pathways	Treatment of osteosarcoma cells with metformin induces cell cycle arrest and apoptosis by modulating ROS dependent JNK/c-Jun cascade.
Chen et al., 2015 [66]	China	Osteosarcoma	MMP2 and MMP9	Treatment with metformin inhibited osteosarcoma cells by inhibiting the formation of cancer stem cell like phenotype via modulating MMP2 and MMP9.
Nguyen et al., 2019 [67]	Republic of Korea	Colorectal cancer	Interleukin 8 (IL-8)	Metformin induced reduction in lithocholic acid-mediated IL8 formation, which in turn inhibited endothelial cell proliferation and tube formation.
Yang et al., 2017 [68]	China	Colorectal cancer	Notch1/Hes1 signaling	In DM2 patients, treatment of metformin inactivated the Notch1/Hes1 signaling axis and reduced the abnormal proliferation of cancer cells.
Liu et al., 2020 [69]	China	Colorectal cancer	Insulin receptor substrate 1 (IRS1)	Metformin treatment of insulin-mediated chemo-resistant cancer cells become sensitive followed by activation pro-apoptotic signaling.
Yang et al., 2025 [70]	China	Bladder, liver, lung and ovarian cancers	Glucose metabolism	Metallodrugs have the potential for effective management of patients with diabetics and cancers.
Qi et al., 2016 [71]	China	Prostate cancer	Glipizide	In hyperglycemic state, glipizide suppresses prostate cancer cells via inhibiting angiogenesis.
Jung et al., 2021 [72]	Republic of Korea	Myocytes	Capmatinib	Anticancer drug, capmatinib could halt insulin resistance in myocytes.
Parada et al., 2019 [73]	USA	Breast cancer	Genetic polymorphism	Genetic polymorphisms associated with diabetics also correlated with the development of breast cancer and cancer related morbidity.
Pechlivanis et al., 2007 [74]	Germany	Colorectal cancer	Insulin receptor substrates	Modulation of insulin resistance pathway genes may associate with the increased risk of colorectal cancer pathogenesis.
Hu et al., 2022 [75]	China	Oral squamous cell carcinoma	Ki67, BRIP1, E-cadherin and cleaved caspase-3	Hyperglycemia affects oral squamous cell carcinoma by modulating cellular proliferation, migration and apoptosis by regulating the expression Ki67, BRIP1, E-cadherin and cleaved caspase-3.

## Data Availability

No new data were created or analyzed in this study. Data sharing is not applicable to this article.

## Supplementary Materials

The following supporting information can be downloaded at: https://www.mdpi.com/article/10.3390/cimb48101086/s1.
