# Supplementary material for: Diabetes and Cancer-Pathophysiology and Clinical Implications: A Systematic Review
Source: Curr Issues Mol Biol. 2026 Oct 20;48(10):1086. doi: 10.3390/cimb48101086 (PMC13703320; doi:10.3390/cimb48101086)
Supplement: Supplementary file 1 [file cimb-48-01086-s001.zip › cimb-4573739-supplementary.pdf]

Contextual References

1. Xu Q, Wu N, Li X, Guo C, Li C, Jiang B, et al. Inhibition of PTP1B blocks pancreatic cancer progression by targeting the PKM2/AMPK/mTORC1 pathway. Cell Death Dis. 2019;10:874. doi:10.1038/s41419-019-2073-4.
2. Hu Y, Zeng N, Ge Y, Wang D, Qin X, Zhang W, et al. Identification of the shared gene signatures and biological mechanism in type 2 diabetes and pancreatic cancer. Front Endocrinol. 2022;13:847760. doi:10.3389/fendo.2022.847760.
3. Pang W, Yao W, Dai X, Zhang A, Hou L, Wang L, et al. Pancreatic cancer-derived exosomal microRNA-19a induces β-cell dysfunction by targeting ADCY1 and EPAC2. Int J Biol Sci. 2021;17:3622-33. doi:10.7150/ijbs.56271.
4. Lu Y, Rodríguez LAG, Malgerud L, González-Pérez A, Martín-Pérez M, Lagergren J, et al. New-onset type 2 diabetes, elevated HbA1c, anti-diabetic medications, and risk of pancreatic cancer. Br J Cancer. 2015;113:1607-14. doi:10.1038/bjc.2015.353.
5. Li C, Sun S, Tu Y, Zhang H, Yao F, Liao S, et al. High glucose accelerates tumor progression by regulating MEDAG-mediated autophagy levels in breast cancer. Int J Biol Sci. 2022;18:4289-300. doi:10.7150/ijbs.70002.
6. Rosendahl AH, Bergqvist M, Lettiero B, Kimbung S, Borgquist S. Adipocytes and obesity-related conditions jointly promote breast cancer cell growth and motility: associations with CAP1 for prognosis. Front Endocrinol. 2018;9:689. doi:10.3389/fendo.2018.00689.
7. Lu H, Yu X, Xu Z, Deng J, Zhang MJ, Zhang Y, et al. Prognostic value of IGFBP6 in breast cancer: focus on glucometabolism. Technol Cancer Res Treat. 2024;23:15330338241271998. doi:10.1177/15330338241271998.
8. Muoio MG, Pellegrino M, Rapicavoli V, Talia M, Scavo G, Sergi V, et al. RAGE inhibition blunts insulin-induced oncogenic signals in breast cancer. Breast Cancer Res. 2023;25:84. doi:10.1186/s13058-023-01686-5.
9. Quail, D.F.; Olson, O.C.; Bhardwaj, P.; Walsh, L.A.; Akkari, L.; Quick, M.L.; Chen, I.-C.; Wendel, N.; Ben-Chetrit, N.; Walker, J.; et al. Obesity Alters the Lung Myeloid Cell Landscape to Enhance Breast Cancer Metastasis through IL5 and GM-CSF. Nat Cell Biol 2017, 19, 974–987, doi:10.1038/ncb3578.
10. Liu H, Xu YT, Li HY, Zhao J, Zhai HY, Chen Y. Loss of microRNA expression is involved in the development and prognosis of breast cancer complicated by type 2 diabetes mellitus. Int J Biol Markers. 2016;31:368-74. doi:10.5301/jbm.5000220.
11. Zhang S, Guo LJ, Zhang G, Wang LL, Hao S, Gao B, et al. Roles of microRNA-124a and microRNA-30d in breast cancer patients with type 2 diabetes mellitus. Tumour Biol. 2016;37:11057-63. doi:10.1007/s13277-016-4981-6.
12. Li Z, Min L, Chen L, Hu Y, Luan W, Li C, et al. Therapeutic potential of anti-miR29a in breast cancer patients with type 2 diabetes: an in vitro and xenograft mouse-model study. Transl Cancer Res. 2022;11:1285-96.

13. Lutz SZ, Hennenlotter J, Scharpf MO, Sailer C, Fritsche L, Schmid V, et al. Androgen receptor overexpression in prostate cancer in type 2 diabetes. Mol Metab. 2018;8:158-66. doi:10.1016/j.molmet.2017.11.013.
14. Teng JA, Wu SG, Chen JX, Li Q, Peng F, Zhu Z, et al. The activation of ERK1/2 and JNK MAPK signaling by insulin/IGF-1 is responsible for the development of colon cancer with type 2 diabetes mellitus. PLoS One. 2016;11:e0149822. doi:10.1371/journal.pone.0149822.
15. Ding J, Zhu YT, Yang L, Tang J, Wang YY, Chen Y, et al. 14-3-3zeta is involved in the anticancer effect of metformin in colorectal carcinoma. Carcinogenesis. 2018;39:493-502. doi:10.1093/carcin/bgy008.
16. Garranzo-Asensio M, Solís-Fernández G, Montero-Calle A, García-Martínez JM, Fiuza MC, Pallares P, et al. Seroreactivity against tyrosine phosphatase PTPRN links type 2 diabetes and colorectal cancer and identifies a potential diagnostic and therapeutic target. Diabetes. 2022;71:497-510. doi:10.2337/db20-1206.
17. Lv X, Ding P, Li L, Li L, Zhou D, Wang X, et al. Increased α-HB links colorectal cancer and diabetes by potentiating NF-κB signaling. Mol Metab. 2023;75:101766. doi:10.1016/j.molmet.2023.101766.
18. Wence-Chavez LI, Palomares-Chacon U, Flores-Gutierrez JP, Jave-Suarez F, Flores-Martinez E, Sanchez-Corona J, et al. Gene expression profiling demonstrates WNT/β-catenin pathway genes alteration in Mexican patients with colorectal cancer and diabetes mellitus. J BUON. 2017;22:1107-14.
19. Bosek I, Sulich A, Rabijewsk MR. Levels of interleukin-2 in patients with colon cancer and diabetes type 2. J Pre Clin Clin Res. 2016;10:1-5. doi:10.5604/18982395.1201825.
20. Mosaad H, Shalaby S, Mahmoud N, Ahmed M, Fayed A, Ashour H, et al. LncRNA ANRIL promotes glucose metabolism and proliferation of colon cancer in a high-glucose environment and is associated with worse outcome in diabetic colon cancer patients. Asian Pac J Cancer Prev. 2024;25:1371-81. doi:10.31557/APJCP.2024.25.4.1371.
21. Papadatos SS, Mitselou A, Lampri E, Bali C, Betzou A, Varouktsi A, et al. Expression of ten-eleven translocation 2 and glutathione-S-transferase pi in colorectal cancer patients with and without type 2 diabetes mellitus. Folia Med. 2024;66:370-9. doi:10.3897/folmed.66.e122713.
22. Li J, Xu J, Cao Z, Du S, Zhang L. MiR-1231 decrease the risk of cancer-related mortality in patients combined with non-small cell lung cancer and diabetes mellitus. Cancer Cell Int. 2020;20:438. doi:10.1186/s12935-020-01525-z.
23. Thamizhanambi TP, Rameshkumar A, Ramya R, Krishnan R, Dineshkumar T, Nandhini G. Analysis of metabolic regulators PGC1-α and PGC1-β in oral squamous cell carcinoma with and without hyperglycemia. Asian Pac J Cancer Prev. 2022;23:2797-803. doi:10.31557/APJCP.2022.23.8.2797.

24. Azimudin R, Palati S, R P. Evaluating tumor necrosis factor (TNF)-alpha pro-inflammatory cytokines in healthy and oral squamous cell carcinoma patients with and without diabetes. Cureus. 2024. doi:10.7759/cureus.52890.
25. Chen K, Wang G, Hu JC, Zhou YY, Ma HT. Clinical significance of Ki-67 in patients with lung adenocarcinoma in situ complicated by type 2 diabetes. World J Diabetes. 2025;16(2):98423. doi:10.4239/wjd.v16.i2.98423.
26. Nishida M, Yamashita N, Ogawa T, Koseki K, Warabi E, Ohue T, et al. Mitochondrial reactive oxygen species trigger metformin-dependent antitumor immunity via activation of Nrf2/mTORC1/P62 axis in tumor-infiltrating CD8+ T lymphocytes. J Immunother Cancer. 2021;9:e002954. doi:10.1136/jitc-2021-002954.
27. Hart PC, Kenny HA, Grassl N, Watters KM, Litchfield LM, Coscia F, et al. Mesothelial cell HIF1α expression is metabolically downregulated by metformin to prevent oncogenic tumor-stromal crosstalk. Cell Rep. 2019;29:4086-98.e6. doi:10.1016/j.celrep.2019.11.079.
28. Hart PC, Chiyoda T, Liu X, Weigert M, Curtis M, Chiang CY, et al. SPHK1 is a novel target of metformin in ovarian cancer. Mol Cancer Res. 2019;17:870-81. doi:10.1158/1541-7786.MCR-18-0409.
29. Pabona JMP, Burnett AF, Brown DM, Quick CM, Simmen FA, Montales MTE, et al. Metformin promotes anti-tumor biomarkers in human endometrial cancer cells. Reprod Sci. 2020;27:267-77. doi:10.1007/s43032-019-00019-2.
30. Kim J, Lee J, Jang SY, Kim C, Choi Y, Kim A. Anticancer effect of metformin on estrogen receptor-positive and tamoxifen-resistant breast cancer cell lines. Oncol Rep. 2016;35:2553-60. doi:10.3892/or.2016.4675.
31. Kang S, Kim BR, Kang MH, Kim DY, Lee DH, Oh SC, et al. Anti-metastatic effect of metformin via repression of interleukin 6-induced epithelial-mesenchymal transition in human colon cancer cells. PLoS One. 2018;13:e0205449. doi:10.1371/journal.pone.0205449.
32. Chen H, Zhao J, Jiang N, Wang Z, Liu C. Hyperglycemia promotes pancreatic cancer initiation and progression by activating the Wnt/β-catenin signaling pathway. Anticancer Agents Med Chem. 2021;21:2592-602. doi:10.2174/1871520621666210201095613.
33. Velazquez-Torres G, Fuentes-Mattei E, Choi HH, Yeung SCJ, Meng X, Lee MH. Diabetes mellitus type 2 drives metabolic reprogramming to promote pancreatic cancer growth. Gastroenterol Rep. 2020;8:261-76. doi:10.1093/gastro/goaa018.
34. Rahn S, Zimmermann V, Viol F, Knaack H, Stemmer K, Peters L, et al. Diabetes as risk factor for pancreatic cancer: hyperglycemia promotes epithelial-mesenchymal-transition and stem cell properties in pancreatic ductal epithelial cells. Cancer Lett. 2018;415:129-50. doi:10.1016/j.canlet.2017.12.004.

35. Parajuli P, Nguyen TL, Prunier C, Razzaque MS, Xu K, Atfi A. Pancreatic cancer triggers diabetes through TGF-β-mediated selective depletion of islet β-cells. Life Sci Alliance. 2020;3:e201900573. doi:10.26508/lsa.201900573.
36. Melloni M, Sergi D, Passaro A, Neri LM. Breast cancer cell line-specific responses to insulin: effects on proliferation and migration. Int J Mol Sci. 2025;26:7523. doi:10.3390/ijms26157523.
37. Strømland PP, Bertelsen BE, Viste K, Chatziioannou AC, Bellerba F, Robinot N, et al. Effects of metformin on transcriptomic and metabolomic profiles in breast cancer survivors enrolled in the randomized placebo-controlled MetBreCS trial. Sci Rep. 2025;15:16897. doi:10.1038/s41598-025-01705-9.
38. Wintrob ZAP, Hammel JP, Khoury T, Nimako GK, Fu HW, Fayazi ZS, et al. Insulin use, adipokine profiles and breast cancer prognosis. Cytokine. 2017;89:45-61. doi:10.1016/j.cyto.2016.10.017.
39. Broggi G, Lo Giudice A, Di Mauro M, Pricoco E, Piombino E, Ferro M, et al. Insulin signaling, androgen receptor and PSMA immunohistochemical analysis by semi-automated tissue microarray in prostate cancer with diabetes (DIAMOND study). Transl Res. 2021;238:25-35. doi:10.1016/j.trsl.2021.07.002.
40. Liu Q, Zhang Y, Wang P, Liu J, Li B, Yu Y, et al. Deciphering the scalene association among type-2 diabetes mellitus, prostate cancer, and chronic myeloid leukemia via enrichment analysis of disease-gene network. Cancer Med. 2019;8:2268-77. doi:10.1002/cam4.1845.
41. Wu Q, Du X, Cheng J, Qi X, Liu H, Lv X, et al. PECAM-1 drives β-catenin-mediated EndMT via internalization in colon cancer with diabetes mellitus. Cell Commun Signal. 2023;21:203. doi:10.1186/s12964-023-01193-2.
42. Mroueh FM, Noureldein M, Zeidan YH, Boutary S, Irani SAM, Eid S, et al. Unmasking the interplay between mTOR and Nox4: novel insights into the mechanism connecting diabetes and cancer. FASEB J. 2019;33:14051-66. doi:10.1096/fj.201900396RR.
43. Zhang L, Zhuo S, Wei X, Song L, Huang S, Xu Y, et al. Epidemiological trends and risk factors of early-onset colorectal cancer in the founding BRICS countries, 1990-2023, with projections to 2050. Front Oncol. 2026;16. doi:10.3389/fonc.2026.1924548.
44. Chatterjee A, Roy T, Swarnakar S. Transcriptional upregulation of MMP-9 gene under hyperglycemic conditions in AGS cells: role of AP-1 transcription factor. Cell Signal. 2024;124:111435. doi:10.1016/j.cellsig.2024.111435.
45. Luo J, Hendryx M, Dong Y. Sodium-glucose cotransporter 2 (SGLT2) inhibitors and non-small cell lung cancer survival. Br J Cancer. 2023;128:1541-7. doi:10.1038/s41416-023-02177-2.
46. Gutkind JS, Molinolo AA, Wu X, Wang Z, Nachmanson D, Harismendy O, et al. Inhibition of mTOR signaling and clinical activity of metformin in oral

premalignant lesions. JCI Insight. 2021;6:e147096. doi:10.1172/jci.insight.147096.

47. Li B, Zhou P, Xu K, Chen T, Jiao J, Wei H, et al. Metformin induces cell cycle arrest, apoptosis and autophagy through ROS/JNK signaling pathway in human osteosarcoma. Int J Biol Sci. 2020;16:74-84. doi:10.7150/ijbs.33787.
48. Chen X, Hu C, Zhang W, Shen Y, Wang J, Hu F, Yu P. Metformin inhibits the proliferation, metastasis, and cancer stem-like sphere formation in osteosarcoma MG63 cells in vitro. Tumour Biol. 2015;36:9873-83. doi:10.1007/s13277-015-3751-1.
49. Nguyen TT, Ung TT, Li S, Lian S, Xia Y, Park SY, Do Jung Y. Metformin inhibits lithocholic acid-induced interleukin 8 upregulation in colorectal cancer cells by suppressing ROS production and NF-κB activity. Sci Rep. 2019;9:2003. doi:10.1038/s41598-019-38778-2.
50. Yang B, Huang CZ, Yu T, Zhou SN, Liu Q, Liu GJ, Chen S, Han FH. Metformin depresses overactivated Notch1/Hes1 signaling in colorectal cancer patients with type 2 diabetes mellitus. Anticancer Drugs. 2017;28:531-9. doi:10.1097/CAD.0000000000000483.
51. Liu C, Liu Q, Yan A, Chang H, Ding Y, Tao J, Qiao C. Metformin revert insulin-induced oxaliplatin resistance by activating mitochondrial apoptosis pathway in human colon cancer HCT116 cells. Cancer Med. 2020;9:3875-84. doi:10.1002/cam4.3029.
52. Yang K, Yang QY, Yang KJ, Hou WY, Chen J, Chen Q, et al. Oxoaporphine-metformin rhodium(III) mixed complex achieving dual antidiabetes and anticancer effects by interfering with glucose metabolism reprogramming. J Am Chem Soc. 2025;147:45145-56. doi:10.1021/jacs.5c14175.
53. Qi C, Li B, Yang Y, Yang Y, Li J, Zhou Q, et al. Glipizide suppresses prostate cancer progression in the TRAMP model by inhibiting angiogenesis. Sci Rep. 2016;6:27819. doi:10.1038/srep27819.
54. Jung TW, Lee HJ, Pyun DH, Kim TJ, Bang JS, Song JH, et al. Capmatinib improves insulin sensitivity and inflammation in palmitate-treated C2C12 myocytes through the PPARδ/P38-dependent pathway. Mol Cell Endocrinol. 2021;534:111364. doi:10.1016/j.mce.2021.111364.
55. Parada H, Cleveland RJ, North KE, Stevens J, Teitelbaum SL, Neugut AI, et al. Genetic polymorphisms of diabetes-related genes, their interaction with diabetes status, and breast cancer incidence and mortality: the Long Island Breast Cancer Study Project. Mol Carcinog. 2019;58:436-46. doi:10.1002/mc.22940.
56. Pechlivanis S, Pardini B, Bermejo JL, Wagner K, Naccarati A, Vodickova L, et al. Insulin pathway related genes and risk of colorectal cancer: INSR promoter polymorphism shows a protective effect. Endocr Relat Cancer. 2007;14:733-40. doi:10.1677/ERC-07-0107.

57. Hu X, Wu J, Xiong H, Zeng L, Wang Z, Wang C, et al. Type 2 diabetes mellitus promotes the proliferation, metastasis, and suppresses the apoptosis in oral squamous cell carcinoma. J Oral Pathol Med. 2022;51:483-92. doi:10.1111/jop.13244.

Table S1. Risk of bias assessment

References	Study	Tool	Provisional rating
19	Xu 2019	SYRCLE (animal) / in vitro checklist	Low
20	Hu 2022	No standard tool; checklist for in silico work	Low
21	Pang 2021	SYRCLE / in vitro checklist	Moderate
22	Lu 2015	ROBINS-E	Low
23	Li 2022	In vitro checklist	Low
24	Rosendahl 2018	ROBINS-E (clinical part); in vitro checklist	Moderate
25	Lu H 2024	ROBINS-E	Moderate
26	Muoio 2023	SYRCLE / in vitro checklist	Low
27	Quail 2019	SYRCLE	Low
28	Liu 2016	ROBINS-E	Low
29	Zhang 2016	ROBINS-E	Moderate
30	Li 2021 (ref. 2022)	SYRCLE / in vitro checklist	Low
31	Lutz 2018	ROBINS-E	Moderate
32	Teng 2016	In vitro checklist	Low
33	Ding 2018	ROBINS-E (tissue); in vitro checklist	Moderate
34	Garranzo-Asensio 2022	ROBINS-E	Moderate
35	Lv 2023	ROBINS-E (patients); SYRCLE (animal)	Moderate
36	Wence-Chavez 2017	ROBINS-E	Low
37	Bosek 2016	ROBINS-E	Low
38	Mosaad 2024	ROBINS-E	Low
39	Papadatos 2024	ROBINS-E	Low
40	Li 2020 (miR-1231)	ROBINS-E	Moderate
41	Thamizhanambi 2022	ROBINS-E	Low
42	Azimudin 2024	ROBINS-E	Moderate
43	Chen 2025	ROBINS-E	Low
44	Nishida 2021	SYRCLE (mouse); ROBINS-E (patients)	Low
45	Hart 2019a	SYRCLE / in vitro checklist	Low
46	Hart 2019b	SYRCLE / in vitro checklist	Low
47	Pabona 2020	In vitro checklist	Low
48	Kim 2016	In vitro checklist	Low
49	Kang 2018	In vitro checklist	Low
50	Chen 2021	SYRCLE / in vitro checklist	Low
51	Velazquez-Torres 2020	SYRCLE	Low
52	Rahn 2018	In vitro checklist	Low
53	Parajuli 2020	SYRCLE	Low
54	Melloni 2025	In vitro checklist	Low

56	Strømland 2025	RoB 2	Low
57	Wintrob 2017	ROBINS-E	Moderate
58	Broggi 2021	ROBINS-E	Low
59	Liu 2019	No standard tool; checklist for in silico work	Low
60	Wu 2023	SYRCLE / in vitro checklist	Low
61	Mroueh 2019	SYRCLE / in vitro checklist	Low
62	Zhang 2026	ROBINS-E adapted / NOS (not ideal)	Moderate
63	Chatterjee 2024	In vitro checklist	Low
64	Luo 2023	ROBINS-E	Moderate
65	Gutkind 2021	RoB 2 if eligible	Low
66	Li 2020 (osteosarcoma)	In vitro checklist	Low
67	Chen 2015	In vitro checklist	Low
68	Nguyen 2019	In vitro checklist	Low
69	Yang 2017	ROBINS-E	Moderate
70	Liu 2020	In vitro checklist	Low
71	Yang 2025	In vitro/in vivo checklist	Low
72	Qi 2016	SYRCLE	Low
73	Jung 2021	In vitro checklist	Low
76	Parada 2019	ROBINS-E	Moderate
85	Pechlivanis 2007	ROBINS-E	Moderate
88	Hu 2022 (OSCC)	In vitro checklist; ROBINS-E (patients)	Low

Scores: High = likely high risk of bias; Moderate= Moderate risk of bias; Low = No or very little risk of bias.
